# Characterizing systemic physiological effects on the blood oxygen level dependent signal of resting‐state fMRI in time‐frequency space using wavelets

**DOI:** 10.1002/hbm.26533

**Published:** 2023-11-11

**Authors:** Quimby N. Lee, Jingyuan E. Chen, Gregory J. Wheeler, Audrey P. Fan

**Affiliations:** ^1^ Department of Neurology University of California‐Davis, School of Medicine Davis California USA; ^2^ Athinoula A. Martinos Center for Biomedical Imaging Massachusetts General Hospital Boston Massachusetts USA; ^3^ Department of Radiology Harvard Medical School Boston Massachusetts USA; ^4^ Department of Biomedical Engineering University of California‐Davis Davis California USA

**Keywords:** heart rate variability, human connectome project, respiration volume per time, resting state networks, resting‐state fMRI, wavelet transform coherence

## Abstract

Systemic physiological dynamics, such as heart rate variability (HRV) and respiration volume per time (RVT), are known to account for significant variance in the blood oxygen level dependent (BOLD) signal of resting‐state functional magnetic resonance imaging (rsfMRI). However, synchrony between these cardiorespiratory changes and the BOLD signal could be due to neuronal (i.e., autonomic activity inducing changes in heart rate and respiration) or vascular (i.e., cardiorespiratory activity facilitating hemodynamic changes and thus the BOLD signal) effects and the contributions of these effects may differ spatially, temporally, and spectrally. In this study, we characterize these brain–body dynamics using a wavelet analysis in rapidly sampled rsfMRI data with simultaneous pulse oximetry and respiratory monitoring of the Human Connectome Project. Our time–frequency analysis across resting‐state networks (RSNs) revealed differences in the coherence of the BOLD signal and heartbeat interval (HBI)/RVT dynamics across frequencies, with unique profiles per network. Somatomotor (SMN), visual (VN), and salience (VAN) networks demonstrated the greatest synchrony with both systemic physiological signals when compared to other networks; however, significant coherence was observed in all RSNs regardless of direct autonomic involvement. Our phase analysis revealed distinct frequency profiles of percentage of time with significant coherence between BOLD and systemic physiological signals for different phase offsets across RSNs, suggesting that the phase offset and temporal order of signals varies by frequency. Lastly, our analysis of temporal variability of coherence provides insight on potential influence of autonomic state on brain–body communication. Overall, the novel wavelet analysis enables an efficient characterization of the dynamic relationship between cardiorespiratory activity and the BOLD signal in spatial, temporal, and spectral dimensions to inform our understanding of autonomic states and improve our interpretation of the BOLD signal.

## Graphical abstract

We characterized the dynamic relationship between cardiorespiratory activity and the blood oxygen level dependent (BOLD) signal in spatial, temporal, and spectral dimensions using a wavelet analysis to inform our understanding of autonomic states and improve our interpretation of the BOLD signal. We identified unique frequency profiles for different phase offsets in instances of coherence between the BOLD signal and systemic physiological dynamics.

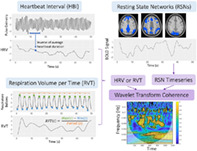

## 1 INTRODUCTION

Resting‐state functional magnetic resonance imaging (rsfMRI) is widely used to investigate resting‐state networks (RSNs), or sets of brain regions that exhibit covarying blood oxygen level dependent (BOLD) signal fluctuations in the absence of explicit stimuli (Biswal et al., 1995). The synchrony between BOLD signals, or functional connectivity, within RSNs is reflective of spontaneous neuronal coactivation between brain regions that support a common function, as demonstrated by studies identifying correlations between simultaneous hemodynamics and neuronal activity across several frequency bands, recorded by intracortical electrophysiology (Shmuel & Leopold, 2008) and calcium imaging (Ma et al., 2016) in animals, and electrocorticography (He et al., 2008; Kucyi et al., 2018; Miller et al., 2009) and magnetoencephalography (Baker et al., 2014; Brookes et al., 2011; Hipp et al., 2012) in humans. Although these studies establish neuronal contributions to functional connectivity, the resting‐state BOLD signal detects changes in blood oxygenation as an indirect measure of neuronal activity and thus can be modulated by hemodynamic and vascular changes driven by autonomic activities, such as heart rate variability (HRV) (Chang et al., 2013) and respiration volume per time (RVT) (Birn et al., 2006). These slow physiological signals oscillate within the same frequency range as neuronally driven hemodynamic fluctuations and are well known to account for a significant variance in fMRI signals during resting state (Shmueli et al., 2007; Wise et al., 2004).

HRV, defined as changes in instantaneous heart rate over time, is a widely used marker of autonomic activity due to its regulation by parasympathetic inhibition and sympathetic excitation (Berntson et al., 1997). Specifically, high frequency (0.15–0.4 Hz) HRV fluctuations are primarily mediated through respiration‐induced heart rate modulation and parasympathetic outflow, while low frequency (0.05–0.15 Hz) HRV fluctuations are facilitated through a mixture of sympathetic and parasympathetic effects. RVT, which measures changes in depth of breathing, can also be modulated by autonomic states, such as vigilance state (Oken et al., 2006) or exercise (Guyenet, 2014), and predominantly fluctuates at 0.02–0.04 Hz at rest due to respiratory feedback mechanisms (Ogoh, 2019). While both of these systemic physiological dynamics are influenced by the central autonomic system, alluding to their neural origins, they also affect blood oxygen delivery (i.e., BOLD) in brain regions through non‐neuronal cerebrovascular modulation, such as changes in blood oxygenation or vascular tone (Birn et al., 2006; Shmueli et al., 2007). Thus, synchrony between regional BOLD activations and systemic physiological dynamics (HRV and RVT) could be due to network‐specific autonomic influences (i.e. brain activity facilitating changes in cardiorespiratory activity) or vascular influences (i.e. cardiorespiratory activity facilitating regional changes in vascular tone and thus the BOLD signal) (Gu et al., 2022).

This synchrony can differ spatially, temporally, and spectrally depending on the relative contributions of neuronal and vascular effects. A previous study differentiated neural and vascular sources of BOLD oscillations at 0.1 Hz by measuring phase‐locking (i.e., changes in phase offsets (Lachaux et al., 1999)) between the BOLD signal and heart rate interval, and identified temporal switching between neuronally and vascularly driven BOLD fluctuations in autonomic brain regions at rest (Pfurtscheller et al., 2017). However, an examination of these systemic physiology‐derived neural and vascular influences of BOLD activation across time, frequency, and brain networks is a fundamental gap in understanding brain–body communication. A spectral characterization of autonomic and vascular contributions to RSNs, with additional phase information to discern temporal lags and ordering, is critical to identifying potential network patterns driven by systemic physiology or cerebrovascular modulation (Bright et al., 2020; Chen et al., 2020) as opposed to neuronal coactivation, and improving functional connectivity interpretations (Glover et al., 2000). Examining the covariance of cardiorespiratory and brain network dynamics can also inform our understanding of the BOLD signal in altered autonomic states, such as autonomic dysregulation in psychiatric disorders (Kemp et al., 2010) or varying arousal states and cardiovascular conditions (Al‐Bachari et al., 2014; Lv et al., 2013). In this study, we will evaluate the coherence between RSN BOLD activations and systemic physiological dynamics at rest in spatial, temporal, and spectral dimensions to characterize autonomic and vascular effects on brain hemodynamics.

Recent studies have suggested that couplings between systemic physiological modulations and the BOLD signal vary across the brain, specifically with spatial organization similar to those of neuronal RSNs. For example, by clustering voxel‐specific physiological response functions across the brain, Chen et al. identified resting‐state “physiological networks” which exhibit distinct BOLD temporal response functions to heartbeat interval (HBI, computed as the instantaneous beat‐to‐beat interval over time) and RVT, and resemble large‐scale functional networks (Chen et al., 2020). Additionally, using a simultaneous hypercapnia challenge orthogonal to cognitive stimuli and subsequent independent component analysis, Bright et al. identified component maps with high spatial overlap with cognitive networks, but dominant temporal correlation with the physiological stimulus (Bright et al., 2020). These studies provide evidence for network‐specific organization of cerebrovascular regulation, which spatially parallels that of neuronal networks and may drive non‐neuronal synchrony between BOLD signals within a network. To further elucidate these potential “physiological networks” and evaluate network‐specific contributions of systemic physiological dynamics to the BOLD signal, in this study, we compare BOLD coherence with HBI/RVT across established RSNs. We hypothesize that there is significant coherence between HBI/RVT and networks traditionally associated with autonomic function (e.g., somatomotor and ventral attention networks) (Chang et al., 2013; Critchley et al., 2003), especially at frequencies where the BOLD signal is predominantly neuronally driven.

To assess the interplay between these biological signals, we utilize wavelet transform coherence (WTC) in rapidly sampled resting‐state fMRI data with simultaneous pulse oximetry and respiratory monitoring of the Human Connectome Project (HCP). WTC is a time–frequency analysis that estimates the linearity and phase difference of two non‐stationary signals with optimized time and frequency resolution (Torrence & Compo, 1998). Previous fMRI studies have used WTC to demonstrate temporal and spectral variability of intra‐ and inter‐network connectivity of the default mode network (Chang & Glover, 2010) and phase offsets between regional brain activations during a visual task (Müller et al., 2004), highlighting dynamic BOLD relationships between brain regions. As opposed to traditional correlation or coherence measures, WTC identifies transient synchrony between non‐stationary BOLD and physiological signals at various frequencies and quantifies phase differences in those instances of synchrony to gain information on the temporal order of the signals. Furthermore, with the fast temporal sampling of the HCP data, we can examine synchronies at higher frequencies of the BOLD signal, where there is emerging evidence of neuronal contributions (Chen & Glover, 2015; Lee et al., 2013; Lewis et al., 2016; Trapp et al., 2018). The wavelet analysis provides a more accurate and efficient method of time–frequency localization for a wider range of dominant frequencies when compared to a conventional windowed Fourier transformation and subsequent correlation analysis (Torrence & Compo, 1998). Overall, by utilizing WTC, we will identify differences in the interplay of systemic physiological signals and BOLD activations across time, frequency, and spatial brain networks.

## 2 METHODS

### 2.1 Data and pre‐processing

Resting‐state fMRI data from 50 healthy young subjects (25 male; 22–35 years old) of the HCP were examined in this study (Smith et al., 2013). Subjects were selected from a cohort that was assessed for peripheral signal quality in a previous study (Chen et al., 2020). For each subject, we analyzed the first 15‐min resting‐state BOLD fMRI session, collected using a gradient‐echo, simultaneous multi‐slice EPI sequence (TR = 0.72 s, TE = 33.1 ms, multi‐band factor = 8, flip angle = 52°, 72 slices, echo spacing 0.58 ms, left‐to‐right phase encoding direction). Peripheral cardiac and respiratory signals were recorded at a 400 Hz sampling rate, using a pulse oximeter placed on the fingertip and a respiratory bellow secured around the chest respectively. Subjects were instructed to keep their eyes open and fixate on a crosshair fixation target for the duration of the scan. All data was previously processed with the “minimal preprocessing pipeline” of the HCP (Glasser et al., 2013), which includes a temporal high‐pass filter of 2000s, but no physiological denoising.

### 2.2 Processing of HBI, RVT, and BOLD timeseries

**FIGURE 1:**
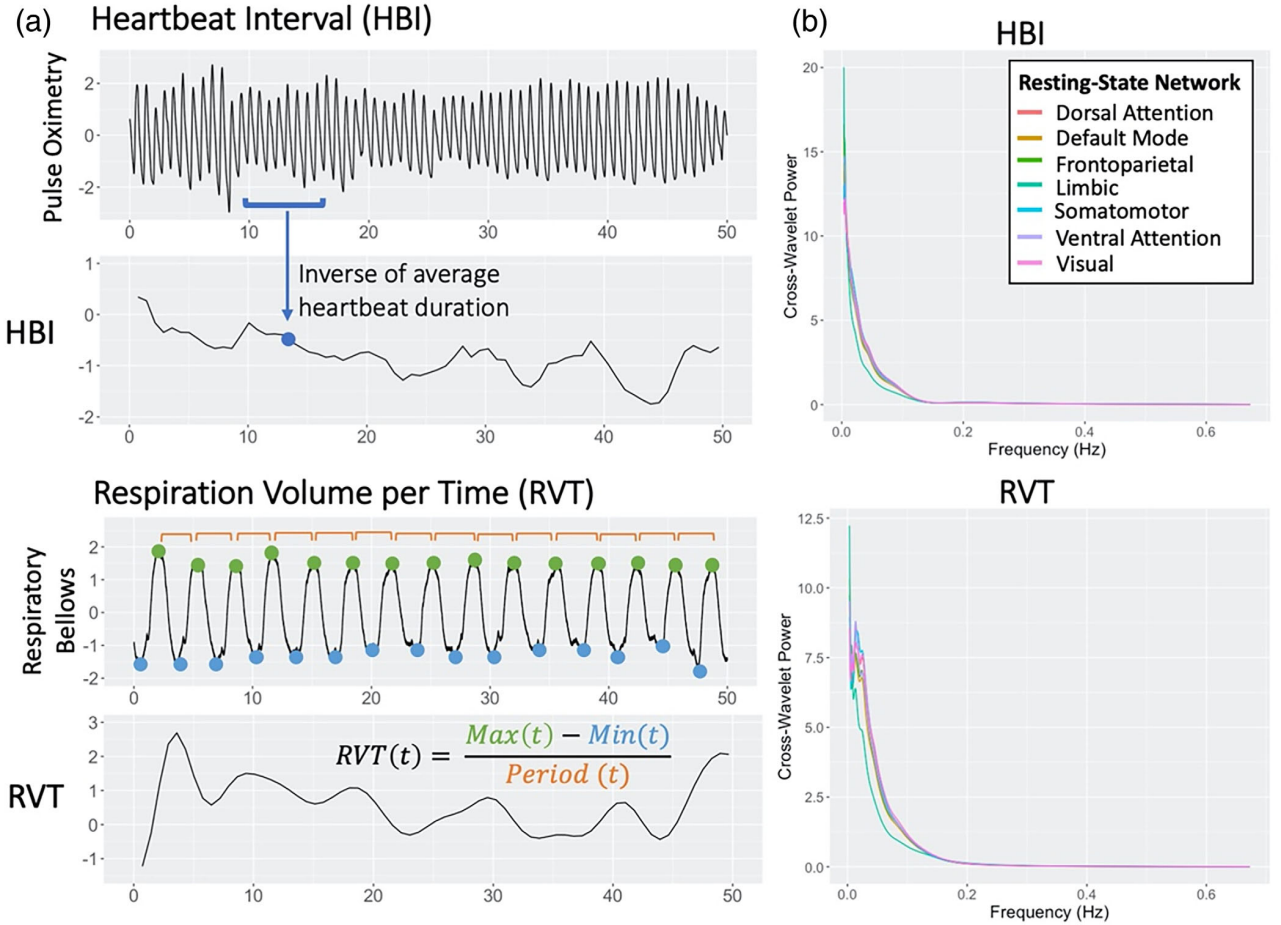
(a) Calculation of heartbeat interval (HBI) and respiration volume per time (RVT) timeseries from raw pulse oximetry or respiratory bellow recordings. (b) Time‐averaged cross‐wavelet power of HBI/RVT and BOLD activations for each resting state network across frequencies.

Peripheral sensor recordings were processed using the TAPAS PhysIO Toolbox (Kasper et al., 2017) of SPM. HBI was calculated as the inverse of average heartbeat durations, measured from pulse oximetry, in 6 s sliding windows centered at the time of each imaging volume (TR). RVT was computed as the difference between inhalation and exhalation amplitudes, measured from respiratory bellows, divided by the temporal spacing of the maxima, and interpolated to the center of each imaging volume (Figure 1a). The average BOLD timeseries was calculated within each of the seven resting‐state networks, including the dorsal attention (DAN), default mode (DMN), frontoparietal (FPN), limbic (LN), somatomotor (SMN), ventral attention (VAN), and visual (VN) networks, identified by Yeo's atlas (Thomas Yeo et al., 2011). Final HBI, RVT, and BOLD RSN timeseries were individually mean‐normalized.

### 2.3 Wavelet transform coherence

**FIGURE 2:**
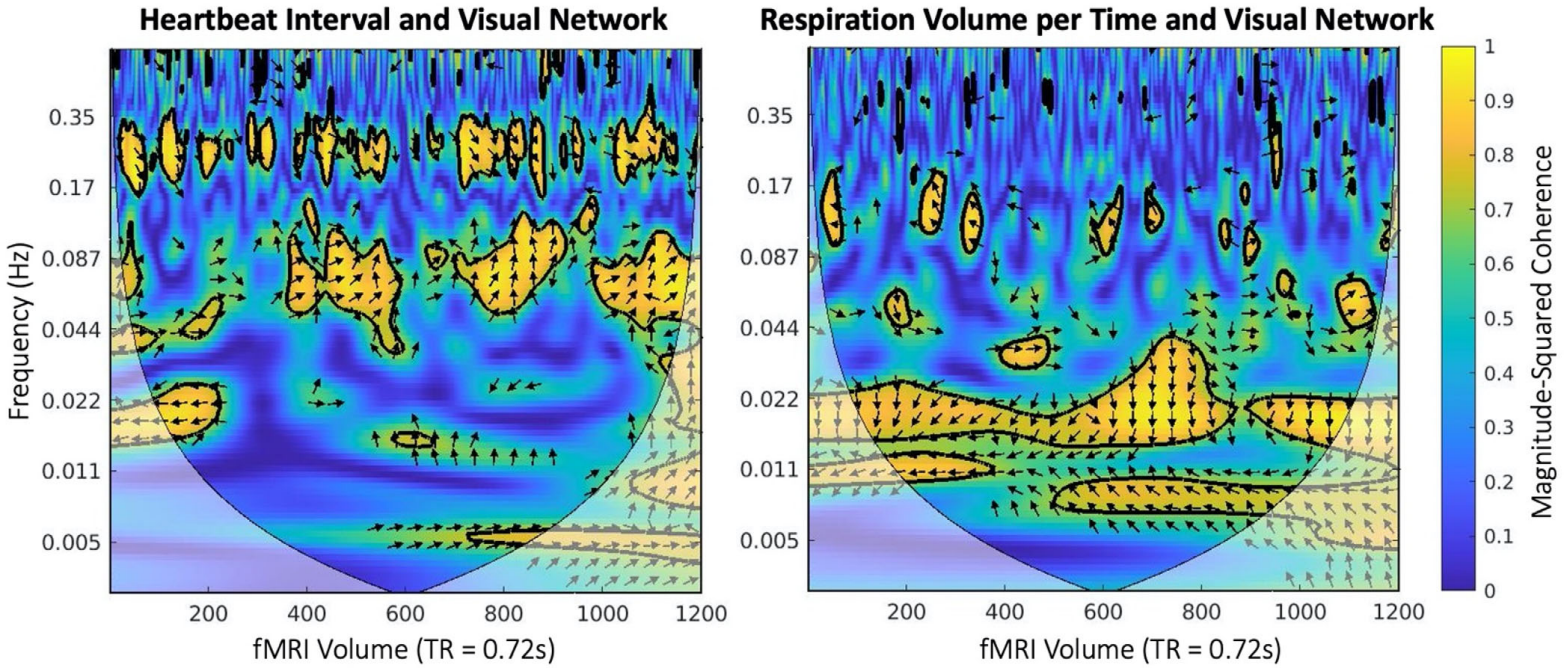
Wavelet transform coherence (WTC) plots from a representative subject. WTC visualization highlights different frequency bands of visual network coherence with heartbeat interval (~0.08 and 0.25 Hz) and respiration volume per time (~0.02 Hz). Color bar signifies magnitude of coherence measured in units of *R* ^2^. Arrows indicate phase offset between the two temporal signals. Outlined areas represent time‐frequency points with significant coherence, as determined by a Monte Carlo simulation with boot‐strapped timeseries.

Wavelet transform coherence (WTC) is a signal‐processing method, based on the continuous wavelet transform (CWT), that measures the coherence and phase lag between two non‐stationary timeseries as a function of both time and frequency. The CWT of a timeseries $x_{n}$ of length N, with uniform time stepsΔt, is defined as:

(1) $$W^{X}\left(n,s\right)=\sqrt{\frac{\Delta t}{s}}\sum_{n^{\prime }=1}^{N}x_{n}\psi_{0}^{*}\left[\left(n^{\prime }-n\right)\left(\frac{∆t}{s}\right)\right],$$

where s represents the wavelet scale. In this study, we define the wavelet function ($\psi_{0}$) as the complex Morlet wavelet:

(2) $$\psi_{0}\left(\eta \right)=\pi^{-1/4}e^{i\omega_{0}\eta }e^{-\eta^{2}/2},$$

with $\omega_{0}=6$, as these parameters provide an optimal trade‐off between time and frequency localization and the Fourier period (T) is approximately equal to the scale s=1.03T (Grinsted et al., 2004; Müller et al., 2004). The CWT captures the power and local phase of a timeseries as a complex value in time and frequency (scale) dimensions. The cross‐wavelet transformation of two timeseries, which is defined as:

(3) $$W^{\mathrm{XY}}\left(n,s\right)=W^{X}\left(n,s\right)W^{Y^{*}}\left(n,s\right),$$

captures the joint power and phase difference between two time series as a function of time and frequency. Cross‐wavelet power is calculated as the modulus, $\left|W^{\mathrm{XY}}\left(n,s\right)\right|$ (Figure 1b). WTC is defined as

(4) $$R^{2}\left(n,s\right)=\frac{{\left|\left\langle s^{-1}W^{\mathrm{XY}}\left(n,s\right)\right\rangle \right|}^{2}}{{\left|\left\langle s^{-1}\left|W^{X}\left(n,s\right)\right|\right\rangle \right|}^{2}\;{\left|\left\langle s^{-1}\left|W^{Y}\left(n,s\right)\right|\right\rangle \right|}^{2}}$$

and produces the magnitude‐squared coherence, or phase‐locked behavior, as an $R^{2}$ value, ranging from 0 to 1 in time and frequency space (Figure 2). Cross‐wavelet power and WTC were calculated between the RSN's average BOLD signal and the peripheral physiological signal for each combination of RSN (DAN, DMN, FPN, LN, SMN, VAN, VN) and peripheral physiological signal (HBI and RVT).

### 2.4 Significance testing of coherence magnitude

A Monte Carlo approach with autoregressive (AR) bootstrapped timeseries was used to evaluate the significance of coherence magnitude. Each input signal (BOLD signal or peripheral physiological signal) was modeled using an AR process of order 1, as used in previous studies to model BOLD timeseries across large regions of interest (Chang & Glover, 2010). In a separate analysis, we also modeled HBI and RVT using a higher model order used in previous studies of HBI (Hipp et al., 2012; Miranda Dantas et al., 2012) and found consistent results to our AR1 analysis (Figures S3 and S4; Table S1). After estimating AR1 coefficients for each signal, 300 pairs of bootstrapped timeseries were generated. WTC magnitude was then calculated for each bootstrapped pair to generate a null distribution and determine a 95% significance threshold at each scale (i.e., frequency).

### 2.5 Group‐level coherence analyses

For each participant, we calculated the percent of timepoints with significant coherence between RSN BOLD and peripheral signal based on the Monte Carlo approach. Percent time with significant coherence was chosen over time‐averaged coherence as our aggregate metric to ensure fair comparisons across frequency, as WTC exhibits different time‐frequency resolution across scales (Chang & Glover, 2010). Values within the “cone of influence” of the WTC time‐frequency space, that is, points where edge‐effects induce lower confidence in calculated values, were excluded from all aggregate coherence metrics. For each dominant frequency band of the respective systemic physiological dynamic (i.e., 0.05–0.15 and 0.15–0.4 Hz for HBI and 0.02–0.04 Hz for RVT), the average percent time with significant coherence across participants was compared between RSNs using repeated measures ANOVAs (RP‐ANOVA). Post‐hoc paired *t*‐tests were performed with Bonferroni correction to correct for multiple comparisons. For each individual RSN, paired *t*‐tests with Bonferroni correction were also used to compare percent time with significant coherence between low (0.05–0.15 Hz) versus high (0.15–0.4 Hz) HBI frequency bands.

Coherence was also measured between RSN signals and a randomly generated timeseries with the same power spectrum as the original HBI/RVT signal (referred to as the null HBI/RVT signal). Null HBI/RVT signals were created by shuffling the phase over time while maintaining the amplitude of the Fourier transform of each signal. The same comparisons between RSNs in each frequency band and between frequency bands for each RSN were performed on RSN coherence with the null HBI/RVT signal to determine if observed network‐specific coherence were due to power differences.

### 2.6 Analysis of phase differences

To further investigate the temporal interplay between these systemic physiological and BOLD signals, points in the time–frequency space were categorized by phase offset ($0\pm \frac{\pi }{4}$, $\frac{\pi }{2}\pm \frac{\pi }{4}$, $\pi \pm \frac{\pi }{4}$, and $-\frac{\pi }{2}\pm \frac{\pi }{4}$) between signals, as calculated by the cross‐wavelet transform. Phase offsets represent temporal lags between two signals as a fraction of the oscillation period, where phase offsets of 0, $\frac{\pi }{2}$, π, and $-\frac{\pi }{2}$ indicate that fluctuations in a timeseries of interest are respectively in‐phase, lagging, anti‐phase, and leading those of a reference signal. In our analysis, we used each RSN BOLD signal as reference and the corresponding HBI/RVT signal as the timeseries of interest, and our interpretations of “BOLD leading” and “HBI/RVT leading” are based on this reference. For each frequency, percent time with significant coherence in each phase category was calculated while again excluding unreliable timepoints inside the cone of influence.

### 2.7 Significance testing of temporal variability of coherence

Our novel wavelet analysis provides a dynamic measure of coherence between two signals, which can inform our physiological interpretation of coherence between RSNs and HBI/RVT. To test whether the observed temporal pattern of coherence between RSNs and HBI/RVT exhibits greater temporal variability than produced by two signals with a stationary relationship, we utilized a Monte Carlo approach with vector autoregressive (VAR) bootstrapped timeseries (Chang & Glover, 2010). Each pair of signals was modeled using a VAR model of order 1. Using the estimated VAR coefficients, 1000 pairs of bootstrapped timeseries were generated. For each bootstrapped pair, WTC was calculated and represented as the complex quantity

(5) $$\mathrm{WTC}\left(n,s\right)=R^{2}\left(n,s\right)\cdot e^{\mathrm{i\phi}\left(n,s\right)},$$

where $\phi \left(n,s\right)$ is the phase. Temporal variability at each scale (s) was calculated as:

(6) $$\mathrm{var}\left(z\right)=<\left(z-\mu_{z}\right){\left(z-\mu_{z}\right)}_{*}>$$

(7) $$\sigma^{2}\left(s\right)=\mathrm{var}\left(\mathrm{WTC}\left(n,s\right)\right)$$

where brackets <> represent the sample mean, * represents the complex conjugate, and $\mu_{z}=<z>$. Unreliable timepoints inside the cone of influence were excluded. For each scale, the null distribution of temporal variability of coherence was used to determine a 95% significance threshold. The percentage of participants with significant temporal variability of coherence was then calculated at each frequency.

## 3 RESULTS

### 3.1 Network‐specific frequency profiles for percent time with significant coherence with HBI/RVT

Both HBI and RVT demonstrated high cross‐wavelet power with RSN signals below 0.1 Hz (Figure 1b). However, individual wavelet transform coherence plots highlight significant coherence between HBI and RSN signals at frequencies above 0.1 Hz (Figure 2). Distinct frequency bands of significant coherence across time are apparent for each peripheral signal and differ by peripheral signal.

**FIGURE 3:**
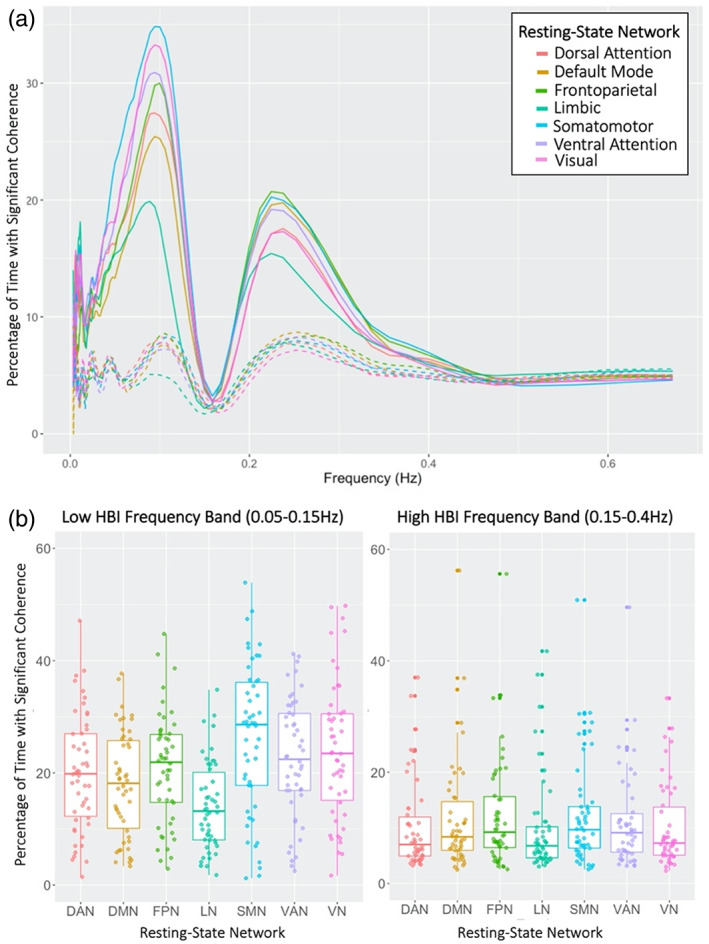
Coherence between heartbeat interval (HBI) and resting‐state network (RSN) blood oxygen level dependent (BOLD) signal activations. (a) Percentage of time with significant coherence between RSN BOLD activations and HBI was averaged across participants for each frequency. Coherence between RSN BOLD activations and a null HBI signal, with the same power spectrum as true HBI, are represented by dashed lines. Standard error bars shown in Figure S1a. (b) Percentage of time with significant coherence was averaged across participants within each frequency band of HBI. RSN profiles were more distinct in the lower frequency band than in the higher frequency band (RP‐ANOVA: low frequency *p* = 6.5e^−17^, high frequency: *p* = 6.0e^−4^; see Table 1 for post‐hoc paired *t*‐test results between RSNs within each frequency band). All RSNs exhibited greater coherence with HBI in the lower frequency band than in the higher frequency band (paired *t*‐test: *p* < .007 for all RSNs).

Percent time with significant coherence with HBI differed by RSN and frequency band. Greater differences in percent time with significant coherence between RSNs were observed in the lower frequency band compared to the higher frequency band of HBI (RP‐ANOVA: low frequency *p* = 6.5e^−17^, high frequency: *p* = 6.0e^−4^). Table 1 details post‐hoc paired *t*‐test results between RSNs within each frequency band. Dominant RSNs also differed by frequency band. At lower frequencies, the SMN, VN, and VAN exhibited the greatest percent time with significant coherence, while at higher frequencies sensorimotor (SMN), salience (VAN), and higher order association networks (DMN and FPN) were dominant (Figure 3b). Percent time with significant coherence was greater in the lower frequency band than in the higher frequency band of HBI for all RSNs (paired *t*‐test: *p* < .007 for all RSNs), which is consistent with low cross‐wavelet power observed at higher frequencies (Figure 1b).

**TABLE 1:** Results of post‐hoc paired *t*‐tests comparing percent time with significant coherence between resting‐state networks (RSN) in each frequency band.

RSN 1	RSN 2	Low frequency HBI (0.05–0.15 Hz)			High frequency HBI (0.15–0.4 Hz)			RVT frequency (0.02–0.04 Hz)		
		*t*‐value	*p*‐value	Sig†	*t*‐value	*p*‐value	Sig†	*t*‐value	*p*‐value	Sig†
DAN	DMN	2.13	3.80E−02	ns	−2.73	.009	ns	1.98	5.40E−02	ns
DAN	FPN	−0.97	3.37E−01	ns	−3.42	.001	*	−0.26	7.95E−01	ns
DAN	LN	5.57	1.08E−06	****	0.59	.561	ns	1.23	2.23E−01	ns
DAN	SMN	−5.56	1.10E−06	****	−4.18	.000118	**	−2.45	1.80E−02	ns
DAN	VAN	−3.00	4.00E−03	ns	−2.34	.023	ns	−4.25	9.65E−05	**
DAN	VN	−5.10	5.49E−06	***	0.14	.886	ns	−0.33	7.45E−01	ns
DMN	FPN	−3.48	1.00E−03	*	−1.68	.099	ns	−3.24	2.00E−03	*
DMN	LN	4.03	1.95E−04	**	3.21	.002	*	−1.33	1.90E−01	ns
DMN	SMN	−6.00	2.32E−07	****	−0.85	.4	ns	−3.56	8.33E−04	*
DMN	VAN	−4.23	1.03E−04	**	0.98	.331	ns	−4.40	5.85E−05	**
DMN	VN	−4.38	6.15E−05	**	2.11	.04	ns	−2.43	1.90E−02	ns
FPN	LN	6.59	2.86E−08	****	3.85	.000344	**	1.88	6.60E−02	ns
FPN	SMN	−4.37	6.55E−05	**	0.16	.874	ns	−1.75	8.60E−02	ns
FPN	VAN	−2.09	4.20E−02	ns	2.19	.033	ns	−3.32	2.00E−03	*
FPN	VN	−3.16	3.00E−03	ns	2.76	.008	ns	−0.05	9.60E−01	ns
LN	SMN	−7.91	2.67E−10	****	−2.98	.004	ns	−3.62	6.90E−04	*
LN	VAN	−7.22	3.02E−09	****	−2.22	.031	ns	−4.73	1.95E−05	***
LN	VN	−7.18	3.44E−09	****	−0.38	.706	ns	−1.49	1.44E−01	ns
SMN	VAN	3.63	6.70E−04	*	2.56	.014	ns	−2.22	3.10E−02	ns
SMN	VN	1.81	7.70E−02	ns	2.56	.014	ns	2.02	4.90E−02	ns
VAN	VN	−1.49	1.41E−01	ns	1.56	.126	ns	3.22	2.00E−03	*

Abbreviations: DAN, dorsal attention network; DMN, default mode network; FPN, frontoparietal network; LN, limbic network; Sig†, significance adjusted with Bonferroni correction; SMN, somatomotor network; VAN, ventral attention network; VN, visual network. *p <0.0024, **p < 2.4E‐4, ***p < 2.4E‐5, ****p < 2.4E‐6

All RSNs were significantly coherent with measured HBI signals for a greater percentage of time than null HBI signals in both the low and high frequency bands of HBI (Figure 3a). When coherence was calculated with a null HBI signal, frequency profiles of percent time with significant coherence did not differ between RSNs (with exception of the LN exhibiting decreased percent time with significant coherence in the lower frequency band), indicating that observed differences in frequency profile between RSNs were not due to their power differences. Percent time with significant coherence between RSN and null HBI signals also did not significantly differ between frequency bands in any of the RSNs, suggesting that the observed differences between frequency bands were not due to power differences.

**FIGURE 4:**
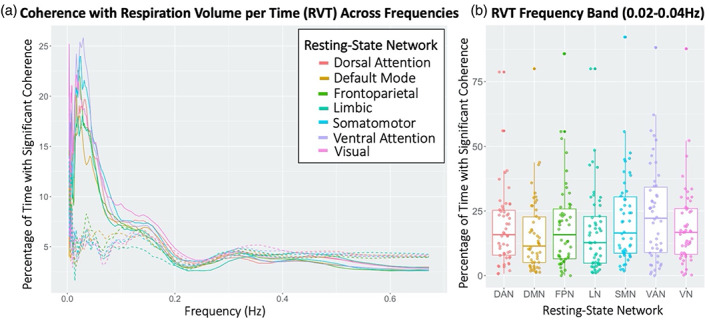
(a) Percentage of time with significant coherence between resting‐state network (RSN) blood oxygen level dependent (BOLD) signal activations and respiration volume per time (RVT) was averaged across participants for each frequency. Coherence between RSN BOLD activations and a null RVT signal, with the same power spectrum as true RVT, are represented by dashed lines. Standard error bars shown in Figure S1b. (b) Percentage of time with significant coherence was averaged across participants within the dominant frequency band of RVT (0.02–0.04 Hz). The ventral attention, somatomotor, and visual network tended to have greater coherence than the default mode, dorsal attention, frontoparietal, and limbic networks (RP‐ANOVA: *p* = 7.3e^−6^; see Table 1 for post‐hoc paired *t*‐test results between RSNs within the RVT frequency band).

For RVT, signals were significantly coherent for the greatest amount of time in the dominant frequency band of RVT, 0.02–0.04 Hz, for all RSNs. However, percent time with significant coherence differed between RSNs within this frequency band (RP‐ANOVA: *p* = 7.3e^−6^; see Table 1 for post‐hoc paired *t*‐test results between RSNs within the RVT frequency band). At these frequencies, percent time with significant coherence was the greatest in the VAN, with sensorimotor (SMN and VN) networks and the FPN also exhibiting significant coherence for larger percentages of time (Figure 4b). Percent time with significant coherence between RSN and null RVT signals did not significantly differ within the RVT frequency band (RP‐ANOVA: *p* = .791), suggesting that observed differences between RSNs were not due to their power differences.

### 3.2 Unique frequency profiles for phase difference categories

**FIGURE 5:**
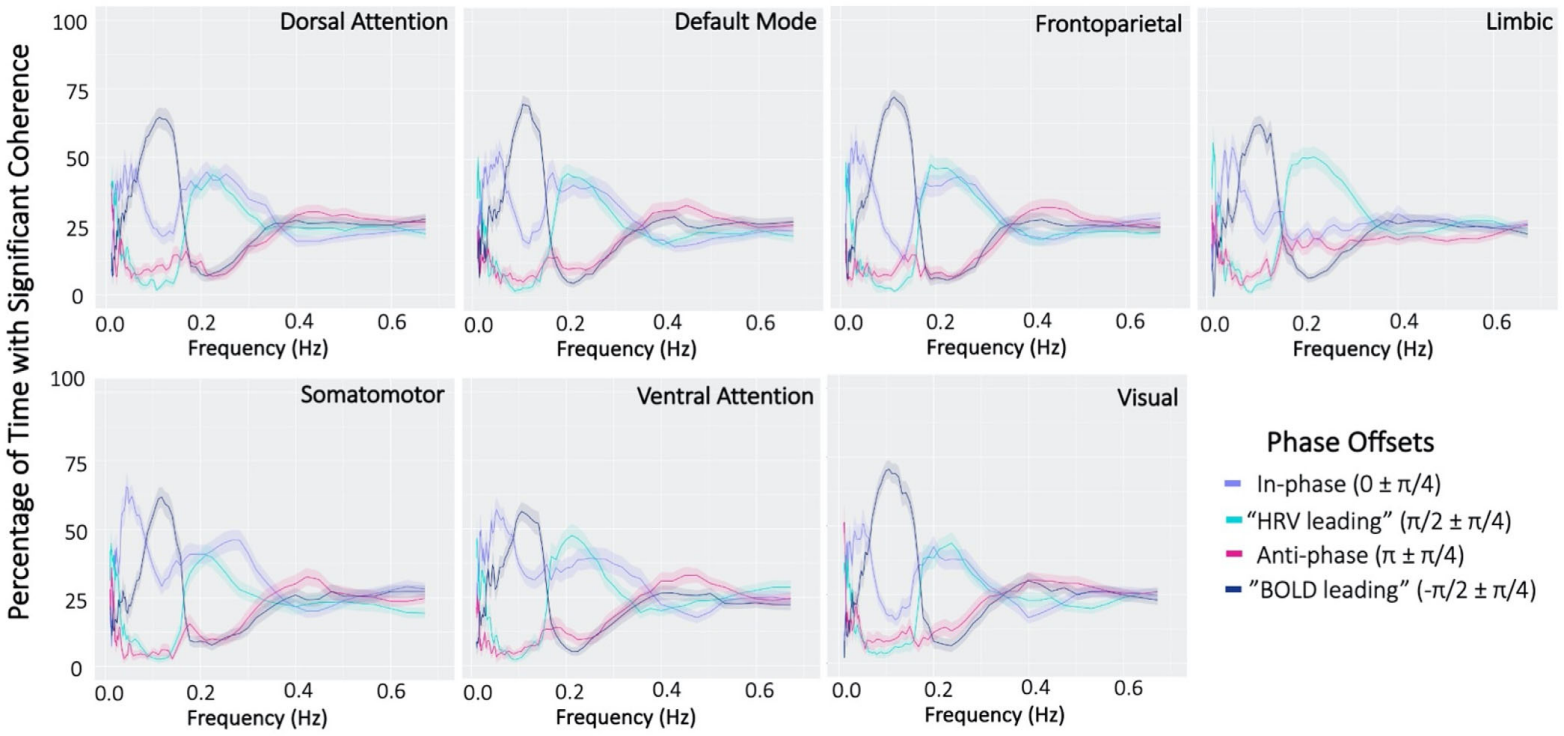
Percentage of time with significant coherence by phase offset for heartbeat interval (HBI) and resting‐state network (RSN) blood oxygen level dependent (BOLD) signal activations. Phase offsets demonstrate distinct frequency profiles across networks. Shaded areas represent standard error.

Across RSNs, phase offset categories demonstrated distinct frequency profiles for percentage of time with significant coherence between HBI and RSN signals (Figure 5). In‐phase instances of coherence ($0\pm \frac{\pi }{4}$) were dominant at lower frequencies (peak ~0.05 Hz), while the anti‐phase category ($\pi \pm \frac{\pi }{4}$) did not exhibit a distinct peak at any frequency. Phase categories that indicate a temporal offset between HBI and RSN signals ($\frac{\pi }{2}\pm \frac{\pi }{4}$ and $-\frac{\pi }{2}\pm \frac{\pi }{4}$), exhibited different frequencies with the greatest percentage of significantly coherent timepoints. The “BOLD leading” group ($-\frac{\pi }{2}\pm \frac{\pi }{4}$) peaked at lower frequencies (~0.15 Hz) and the “HBI leading” group ($\frac{\pi }{2}\pm \frac{\pi }{4}$) at higher frequencies (~0.25 Hz). This observation indicates that temporal order of HBI and BOLD signals varies depending on frequency band. Interestingly, peak frequencies also differed between in‐phase and “BOLD‐leading” phase categories in the lower frequency band (~0.05 and 0.15 Hz respectively). Frequency profiles for each phase category remained consistent across RSNs, with exception of the LN, which lacked a “HBI leading” peak at high frequencies. Overall, our analysis demonstrated that phase offsets between HBI and the BOLD signal differ by frequency.

**FIGURE 6:**
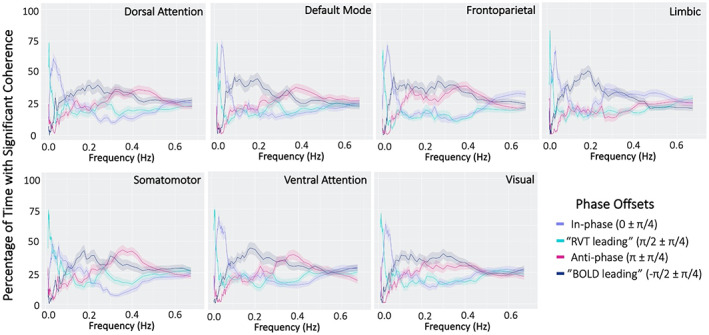
Percentage of time with significant coherence by phase offset for respiration volume per time (RVT) and resting‐state network (RSN) blood oxygen level dependent (BOLD) signal activations. Phase offsets demonstrate distinct frequency profiles across networks. Shaded areas represent standard error.

Dominant frequencies of coherence between RVT and the BOLD signal also differed by phase category across networks (Figure 6). In‐phase instances of coherence ($0\pm \frac{\pi }{4}$) were dominant at lower frequencies (~0.04 Hz), while anti‐phase instances generally represented low percentages of significant timepoints, with a slight peak at higher frequencies (~0.4 Hz). Phase categories that indicate temporal offset between RVT and BOLD signals ($\frac{\pi }{2}\pm \frac{\pi }{4}$and $-\frac{\pi }{2}\pm \frac{\pi }{4}$), again differed in the frequencies where they show the greatest percentage of significant timepoints. While “RVT leading” category ($\frac{\pi }{2}\pm \frac{\pi }{4}$) represents a large percentage of coherent timepoints at very low frequencies (peak ~0.02 Hz), “BOLD leading” instances generally made‐up low percentages of coherent timepoints, with a slight peak at higher frequencies (peak ~0.2 Hz). This indicates that temporal order of RVT and BOLD signals also varies with frequency band. Frequency profiles for in‐phase and “RVT leading” categories remained consistent across RSNs, while the small peaks of anti‐phase and “BOLD leading” categories differed slightly between networks. Overall, our analysis demonstrated that phase offsets between RVT and the BOLD signal differ by frequency.

### 3.3 Network‐specific frequency profiles for percentage of participants with significant temporal variability of coherence with HBI/RVT

**FIGURE 7:**
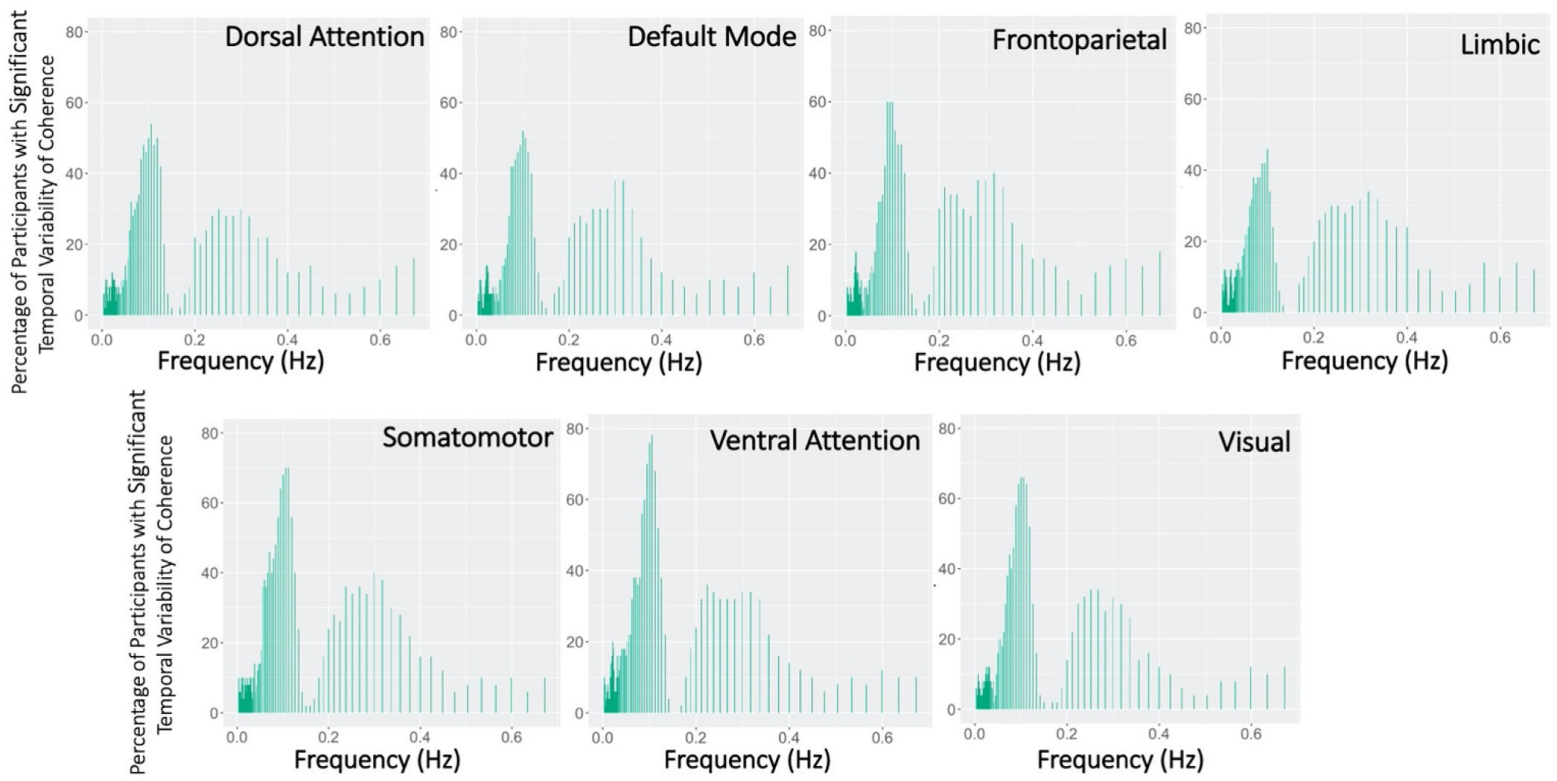
Percentage of participants with significant temporal variability of coherence between heartbeat interval (HBI) and resting‐state network (RSN) blood oxygen level dependent (BOLD) signal activations. Significance of temporal variability at each frequency was determined by a Monte Carlo approach with bootstrapped timeseries generated with a vector autoregressive model.

In all RSNs, the percentage of participants with significant temporal variability of coherence with HBI peaked within the two key frequency bands of HBI. A greater percentage of participants had significant temporal variability of coherence in the lower frequency band (>50%) than the higher frequency band (Figure 7). Peak percentages, especially in the lower frequency band, differed by RSN, with the highest percentage observed in sensory and salience networks (SMN, VN, VAN).

**FIGURE 8:**
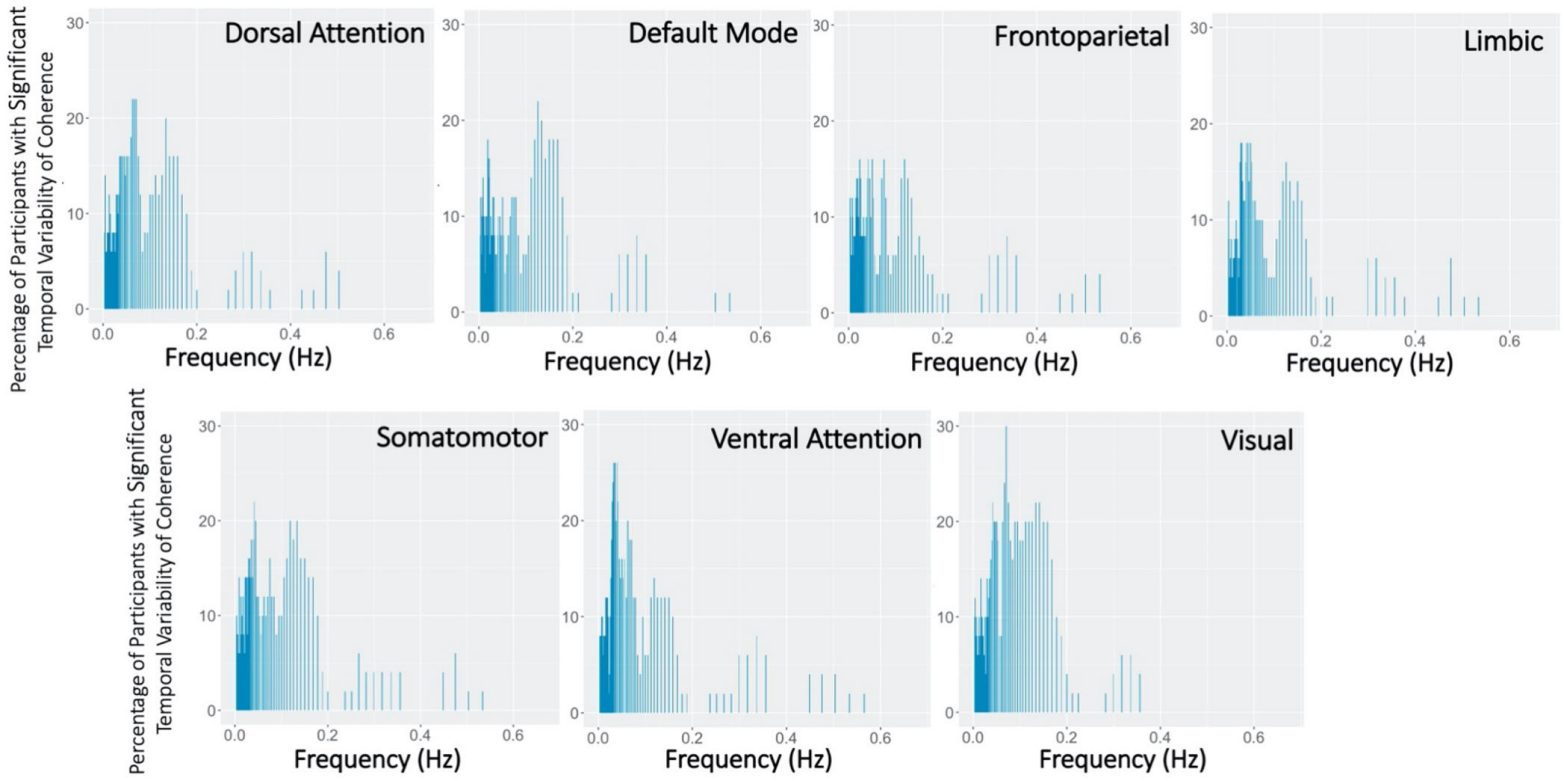
Percentage of participants with significant temporal variability of coherence between respiration volume per time (RVT) and resting‐state network (RSN) blood oxygen level dependent (BOLD) signal activations. Significance of temporal variability at each frequency was determined by a Monte Carlo approach with bootstrapped timeseries generated with a vector autoregressive model.

The percentage of participants with significant temporal variability of coherence with RVT peaked at lower frequencies of RVT in all RSNs. However, the significant temporal variability of coherence was observed in less than half of all participants for all RSNs, fewer than for HBI (Figure 8). Peaks in percentage of participants with significant temporal variability of coherence were observed at about 0.05 and 0.15 Hz in most RSNs.

## 4 DISCUSSION

This study provides a characterization of coherence between BOLD activations and systemic physiological signals across RSNs using a time‐frequency wavelet analysis. We identified higher synchrony between HBI and rsfMRI signals within a low frequency regime of HBI fluctuations (0.05–0.15 Hz), compared to its higher frequency regime (0.15–0.4 Hz), with unique profiles for RSNs. Distinct RSN profiles were also observed in the synchrony between BOLD activations and RVT fluctuations in its dominant frequency band (0.02–0.04 Hz). Somatomotor (SMN), visual (VN), and salience (VAN) networks demonstrated the greatest percent time with significant coherence with both systemic physiological signals when compared to other RSNs, which is consistent with their established roles in autonomic processing. However, significant coherence was observed in all RSNs regardless of direct autonomic involvement. Furthermore, our phase analysis revealed distinct frequency profiles of percentage of time with significant coherence between BOLD and systemic physiological signals for different phase offset categories across RSNs. This observation suggests that the phase offset and temporal order of signals varies by frequency. Lastly, we identified significant temporal variability in coherence for a majority of participants in the lower frequency band of HBI, with distinct frequency profiles across networks. Frequency profiles for percentage of participants with significant temporal variability of coherence with RVT also differed by RSN, but with fewer participants exhibiting significant temporal variability of coherence at all frequencies. Characterizing the phase offset and temporal variability of coherence can help inform our understanding of neuronal (i.e., brain activity facilitating changes in cardiorespiratory activity) and vascular (i.e., cardiorespiratory activity facilitating regional changes in vascular tone and thus the BOLD signal) components of systemic physiological effects on the BOLD signal at rest and in altered autonomic states.

We identified significant coherence between HBI and BOLD activations of all RSNs, with strongest coherence in RSNs involved in somatomotor or salience processing. Our findings are consistent with previous studies that identified significant correlations between HBI dynamics and the BOLD time series in autonomic regions associated with salience processing and motor control, such as the anterior cingulate cortex, putamen, insula, and supplementary motor area, at rest (Chang et al., 2013) and in response to a task (Critchley et al., 2003). This supports the hypothesis that synchrony between HBI and BOLD signals is a result of neuronal activation to salient stimuli eliciting both a local, cerebral hemodynamic modulation (i.e., BOLD change) and a systemic adjustment in physiology (i.e., HBI change). Our phase analysis in the lower frequency band (0.05–0.15 Hz) is consistent with this theory, as HBI and RSN signals tended to be in‐phase or show BOLD leading HBI (based on the $-\frac{\pi }{2}$ category) during coherent timepoints at low frequencies, indicating that changes in BOLD precede changes in HBI. Our analysis of temporal variability also supports this interpretation, as RSN and HBI signals exhibited high temporal variability in their synchrony, especially in the lower frequency band, suggesting that synchrony between signals varies over time. Changes in autonomic state could be one source of this variability, as states of arousal could influence the degree of brain–body communication. Future studies could conduct similar wavelet coherence analyses in altered autonomic states, such as autonomic dysregulation in psychiatric conditions (Kemp et al., 2010; Licht et al., 2008), various vigilance states (Chang et al., 2013), sleep stages (Fultz et al., 2019), and stress (Wang et al., 2005), to gain information on differences in brain–body communication in these conditions.

Despite observing the highest coherence in autonomic‐related RSNs, such as SMN, VAN, and VN, significant coherence with HBI was apparent in both frequency bands for all RSNs, regardless of their traditional association with the autonomic system, which points to wider involvement of RSNs in autonomic activity. A recent study identified significant autonomic associations between BOLD and cardiovagal activity in more diverse regions of the brain than previously reported using correlation of fMRI with inhomogeneous point‐process models of heart beat dynamics in the HCP dataset (Valenza et al., 2019). In particular, this study found classic DMN regions, such as the posterior cingulate cortex, precuneus, anterior cingulate cortex, and medial prefrontal cortex, and FPN regions, such as the middle cingulate cortex, superior frontal gyrus, and superior parietal lobule, to be significantly correlated with heartbeat dynamics, suggesting a dynamic role of the autonomic system. Our findings support this interpretation of the autonomic network as a collection of brain regions that both mediates systemic physiological dynamics and facilitates higher processing, rather than an independent network. Separate from autonomic roles, RSNs may show significant coherence to systemic physiology due to parenchymal microvascular density or proximity to large brain vessels. High vascularization or proximity to large vessels may induce significant coherence due to greater influence of non‐neuronal vascular dynamics on the BOLD signal. However, a previous study comparing brain regions with high cardiorespiratory response function amplitudes and brain regions high vessel density found multiple cases where high BOLD amplitudes did not align with high vessel density, suggesting that vascular anatomy only partially accounts for variation in cardiovascular effects of the BOLD signal across the brain (Chen et al., 2020).

Furthermore, we identified significant synchrony between HBI and RSNs in both low and high frequency bands of HBI. Significance of coherence at higher frequencies could be due to parasympathetic modulations of heart rate, which alter cerebral blood flow, vascular tone, and oxygenation, or remnant quasiperiodic fluctuations from cardiorespiratory cycles (Katura et al., 2006; Shmueli et al., 2007). The phase analysis supports our predominantly vascular interpretation of the synchrony at higher frequencies. At higher frequencies, coherent timepoints exhibit HBI and BOLD signals in‐phase or HBI leading BOLD (based on the $\frac{\pi }{2}$ category), alluding that changes in heart rate induce vascular factors that affect the BOLD signal. When conducting the same analysis on 10 ICA‐FIX (FMRIB's ICA‐based X‐noiseifier) (Salimi‐Khorshidi et al., 2014; Smith et al., 2013) denoised rsfMRI scans of HCP, in which spatial and temporal components classified as noise are regressed out, percentage of time with significant coherence with DMN was overall decreased, especially in the higher frequency band of HBI (Figure S2a). This suggests that coherence in the higher frequency band of HBI is driven by quasiperiodic fluctuations such as respiration, which occupies similar frequencies and modulates heartbeat periods through respiratory sinus arrhythmia (Berntson et al., 1997). However, phase category trends remained the same, with characteristic in‐phase peaks at 0.05 and 0.25 Hz, a “BOLD leading” peak at 0.15 Hz, and a “HBI leading” peak at 0.25 Hz. Future analysis using RETROICOR (Glover et al., 2000) to remove quasiperiodic cardiac and respiratory fluctuations could be performed to further elucidate the contributions of these dynamics to the high frequency BOLD signal.

Lower coherence and greater differences in coherence across RSNs in the higher frequency regime compared to the lower frequency regime are likely due to inherent hemodynamic filtering (i.e., a low‐pass filter) of the BOLD signal. However, recent studies have identified that the BOLD signal can measure neuronal activity at higher frequencies than previously assumed by the canonical hemodynamic response function (Chen & Glover, 2015; Lee et al., 2013; Lewis et al., 2016; Trapp et al., 2018), although the precise mechanisms underlying these fast fMRI dynamics remain unclear. As one possible mechanism, Chen et al. demonstrated that lower intensity stimuli, such as spontaneous neural events at rest (Liu & Duyn, 2013), elicit faster and narrower hemodynamic responses, which enables high frequency fluctuations in neural activity to manifest in the BOLD signal (Chen et al., 2021; Thompson et al., 2014). With this interpretation, higher frequency components of the BOLD signal are likely composed of a variety of small, spontaneous neural dynamics, compared to lower frequency components that are dominated by large, salient neural events. Thus, high frequency BOLD fluctuations may be less coherent with major autonomic changes, such as HBI, due to the high frequency BOLD signal's composition of a variety of smaller, transient neuronal changes. This potential reasoning is consistent with our finding that RSNs have similar synchrony with HBI in the higher frequency band, that is, networks associated with neurogenic autonomic processing have more similar coherence to other networks.

Recently, Attarpour et al. conducted a similar frequency analysis of the coherence between cardiac and regional BOLD signals, focusing specifically in cerebrospinal fluid (CSF) regions (Attarpour et al., 2021). Consistent with our study, they observed peaks in coherence with HBI in both the low and high frequency regimes in all regions, including gray matter, white matter, and CSF. Brain tissues and venous regions exhibited peak coherence at lower frequencies, similar to those identified in gray matter regions in our study, while CSF and arterial ROIs exhibited peak coherence at higher frequencies up to 0.25 Hz. This finding supports our interpretation of high frequency coherence having primarily vascular as opposed to neuronal origins, as CSF oscillations contain essentially no neuronal signals and show dominant coherence at high frequencies. In contrast to our study of gray matter regions, in CSF the frequency of peak coherence varied from 0.14 Hz in the lateral ventricles to higher frequencies up to 0.25 Hz in the third ventricle and cerebral aqueduct. These discrepancies point to differing mechanisms facilitating the relationship between HBI and BOLD activations in gray matter and CSF regions.

We also observed significant synchrony between RVT and the BOLD signal in the dominant frequency band of RVT (0.02–0.04 Hz) for all RSNs. Phase offsets within this frequency band indicate that RVT changes precede BOLD changes (based on the $\frac{\pi }{2}$ category), which points to RVT‐induced changes in blood oxygenation or vascular tone mediating changes in the BOLD signal at these frequencies (Birn et al., 2006). Our analysis of temporal variability also supports this vascular interpretation of BOLD–RVT coherence, as only a minority of participants exhibited significant temporal variability of coherence, even at the key RVT frequencies, indicating that the synchrony between BOLD and RVT signals is consistent over time. When compared to neurogenic effects, vascular changes have a more direct effect on blood oxygenation (i.e., the BOLD signal) and thus the relationship between BOLD and RVT may vary less over time. Furthermore, the coherence magnitude was observed in salience and somatomotor networks. A previous study identified a distinct respiratory response function for sensorimotor regions compared to other gray matter regions (Chen et al., 2020). A respiratory response function characterizes the timing and magnitude of the BOLD changes in response to RVT changes. Thus, differences in the timing between RVT change and BOLD response, such as those observed in somatomotor regions, can lead to greater synchrony of the BOLD and RVT signals in that network. Higher synchrony in somatomotor and salience networks could also reflect vascular brain organization that spatially match neuronal networks and induce network‐specific coherence with systemic physiological signals, such as RVT (Bright et al., 2020; Chen et al., 2020).

While our wavelet analysis provides a unique characterization of instantaneous coherence across time and frequencies, this analysis may not be generalizable to all datasets. In this study, we leveraged the high sampling rate of resting‐state fMRI data provided by the HCP to analyze a large frequency range with reduced effects of signal aliasing. The high temporal resolution of HCP data allowed us to more accurately investigate the relationship between systematic physiology and neuronal signals; however, resting‐state fMRI data with lower temporal resolution may suffer from frequency aliasing and exhibit different patterns of coherence. Modern simultaneous multislice sequences enable sub‐second acquisitions with similar temporal resolutions to our study, thus increasing the generalizability of our findings. Furthermore, analyses of non‐stationary signals, such as our wavelet analysis, may require longer timeseries to disentangle frequency components over time. To test the effect of scan duration on our results, we extracted subsets of the original 15 min of BOLD signal and HBI/RVT recordings into 5‐ and 10‐min timeseries and observed similar frequency profiles and RSN differences across scan durations (Figure S4), suggesting that our analyses and findings are generalizable to datasets with reduced scan duration. RVT results may be less reliable at shorter scan durations due to differences in quality of respiratory recordings.

Caution should also be taken in the interpretations of phase offsets. To interpret temporal order from a phase offset, we must assume that the beginning of each recording represents the definitive start of the signal. However, biological signals, such as HBI, RVT, and BOLD activations, exist before and after our recorded signals, which makes it difficult to conclude which signal is leading or lagging in instances of temporal offset. To help interpret temporal order on a group level, we identified the median time lag with the highest cross‐correlation between HBI/RVT and RSN signals across participants for each frequency band. The positive or negative time lag, in conjunction with phase difference, was then used to estimate temporal order of signals for that frequency band. We observed the highest cross‐correlation between HBI and RSN signals when BOLD led HBI by ~2 s in the lower frequency band and when BOLD lagged HBI by ~1 s in the higher frequency band. The contrasting temporal order of signals is consistent with our findings of opposite phase offsets in the two frequency bands. In the RVT frequency band, we observed the highest cross‐correlation between RVT and RSN signals when BOLD lagged RVT by ~3 s, which is consistent with our interpretation of RVT leading RSN signals.

There are several limitations to our study. First, to calculate BOLD activations per RSN, we averaged the temporal signal across large areas of the Yeo atlas, which decreases region‐specific sensitivity of our BOLD measurements, but increase the signal‐to‐noise ratio of our timeseries. Despite this averaging, we were able to identify significant network differences in coherence, supporting the network‐specificity of our aggregate BOLD signals. Additionally, magnitudes of coherence did not correspond to the size of RSN masks, indicating that observed differences in coherence were not due to differences in the number of voxels averaged. Next, the BOLD signal and our peripheral measurements are recorded at disparate areas of the body, which may influence the coherence and phase offsets between signals. We were able to find significant coherence between timeseries recorded on these separate devices; however, the timing differences of our recording devices is a limitation in interpreting our phase offsets. Lastly, we did not convolve our systemic physiological signals with response functions before coherence analysis to avoid assuming a temporal order of the signals, as applying cardiorespiratory response functions would enforce peripheral signals leading BOLD signals and applying the hemodynamic response function would enforce the reverse. Additionally, there is evidence of differing cardiorespiratory response functions across gray matter (Chen et al., 2020) and applying a singular response function may induce differences in coherence. However, our use of non‐convolved signals may reduce overall coherence between signals, as we do not account for differences in the timing or shape of the response between signals. Despite these concerns, we were still able to identify significant coherence for both signal orderings (i.e., BOLD leading and HBI/RVT leading) and a majority of our time lags are consistent with expected physiological timing.

## 5 CONCLUSION

Ultimately, this study provides insight into the dynamic relationship between systemic cardiovascular modulations and neuronal activity. Utilizing phase offset and temporal variability information provided by our wavelet analysis of coherence, we identified differences in vascular versus autonomic effects across frequencies and RSNs. Disentangling these components of the BOLD signal can inform fMRI methods to investigate these vascular or autonomic dynamics with greater specificity. Further studies using this method could also enhance our understanding of brain–body communication and functional connectivity interpretations in altered autonomic states. In summary, using a wavelet coherence analysis, we were able to characterize synchrony between systemic physiological dynamics and BOLD activations in temporal, spectral, and spatial dimensions to improve autonomic and vascular interpretations of the BOLD signal.

## CONFLICT OF INTEREST STATEMENT

The authors declare no competing financial interests.

## Supporting information


**FIGURE S1:** Percentages of time with significant coherence between blood‐oxygen level dependent (BOLD) signal activations and each systemic physiological dynamic (a) heart rate variability [HRV] and (b) respiration volume per time [RVT]) were averaged across participants for each frequency and resting‐state network (RSN). Coherence between RSN BOLD activations and a null HRV/RVT signal, with the same power spectrum as true HRV/RVT, are represented by dashed lines. Shaded areas represent standard error.
**FIGURE S2.** Coherence profiles after ICA‐FIX denoising to remove quasiperiodic fluctuations in 10 participants. (a) Percentages of time with significant coherence between blood‐oxygen level dependent (BOLD) signal activations in the default mode network (DMN) and each systemic physiological dynamic were averaged across participants for each frequency. Coherence between DMN BOLD activations and a null HRV/RVT signal, with the same power spectrum as true HRV/RVT, are represented by dashed lines. Shaded areas represent standard error. (b) Phase offsets demonstrate distinct frequency profiles across networks. Shaded areas represent standard error.
**FIGURE S3.** Percentages of time with significant coherence between blood‐oxygen level dependent (BOLD) signal activations and heartbeat interval (HBI). Significance testing of coherence magnitude was performed with a Monte Carlo approach with bootstrapped timeseries. BOLD signals and HBI were modeled using autoregressive models of orders 1 and 9 respectively. After estimating AR1 coefficients for each signal, 300 pairs of bootstrapped timeseries were generated. WTC magnitude was then calculated for each bootstrapped pair to generate a null distribution and determine a 95% significance threshold at each scale (i.e. frequency).
**FIGURE S4.** Percentages of time with significant coherence between blood‐oxygen level dependent (BOLD) signal activations and respiration volume per time (RVT). Significance testing of coherence magnitude was performed with a Monte Carlo approach with bootstrapped timeseries. BOLD signals and RVT were modeled using autoregressive models of orders 1 and 9 respectively. After estimating AR1 coefficients for each signal, 300 pairs of bootstrapped timeseries were generated. WTC magnitude was then calculated for each bootstrapped pair to generate a null distribution and determine a 95% significance threshold at each scale (i.e., frequency).
**TABLE S1.** Results of post‐hoc paired *t*‐tests comparing percent time with significant coherence between resting‐state networks (RSN) in each frequency band. Significance testing of coherence magnitude was performed with a Monte Carlo approach with bootstrapped timeseries. BOLD signals and RVT were modeled using autoregressive models of orders 1 and 9 respectively. After estimating AR1 coefficients for each signal, 300 pairs of bootstrapped timeseries were generated. WTC magnitude was then calculated for each bootstrapped pair to generate a null distribution and determine a 95% significance threshold at each scale (i.e., frequency). DAN, dorsal attention network; DMN, default mode network; FPN, frontoparietal network; LN, limbic network; Sig†, significance adjusted with Bonferroni correction; SMN, somatomotor network; VAN, ventral attention network; VN, visual network.
**FIGURE S5.** Coherence between systemic physiological dynamics and resting‐state network (RSN) blood oxygen level dependent (BOLD) signal activations across scan durations. The original 15‐min resting‐state functional MRI scan was truncated into 5‐ and 10‐min segments to assess the reproducibility of our results across scan duration. Wavelet frequencies with greater than 2 min of scan time outside of the unreliable “cone of influence”, in which edge artifacts are problematic, are included.

## Data Availability

The data that support the findings of this study are available from the corresponding author upon reasonable request.
