# Supplementary material for: Characterizing systemic physiological effects on the blood oxygen level dependent signal of resting‐state fMRI in time‐frequency space using wavelets
Source: Hum Brain Mapp. 2023 Nov 11;44(18):6537–51. doi: 10.1002/hbm.26533 (PMC10681653; doi:10.1002/hbm.26533)
Supplement: Supplementary file 1 — FIGURE S1: Percentages of time with significant coherence between blood‐oxygen level dependent (BOLD) signal activations and each systemic physiological dynamic (a) heart rate variability [HRV] and (b) respiration volume per time [RVT]) were averaged across participants for each frequency and resting‐state network (RSN). Coherence between RSN BOLD activations and a null HRV/RVT signal, with the same power spectrum as true HRV/RVT, are represented by dashed lines. Shaded areas represent standard error. FIGURE S2. Coherence profiles after ICA‐FIX denoising to remove quasiperiodic fluctuations in 10 participants. (a) Percentages of time with significant coherence between blood‐oxygen level dependent (BOLD) signal activations in the default mode network (DMN) and each systemic physiological dynamic were averaged across participants for each frequency. Coherence between DMN BOLD activations and a null HRV/RVT signal, with the same power spectrum as true HRV/RVT, are represented by dashed lines. Shaded areas represent standard error. (b) Phase offsets demonstrate distinct frequency profiles across networks. Shaded areas represent standard error. FIGURE S3. Percentages of time with significant coherence between blood‐oxygen level dependent (BOLD) signal activations and heartbeat interval (HBI). Significance testing of coherence magnitude was performed with a Monte Carlo approach with bootstrapped timeseries. BOLD signals and HBI were modeled using autoregressive models of orders 1 and 9 respectively. After estimating AR1 coefficients for each signal, 300 pairs of bootstrapped timeseries were generated. WTC magnitude was then calculated for each bootstrapped pair to generate a null distribution and determine a 95% significance threshold at each scale (i.e. frequency). FIGURE S4. Percentages of time with significant coherence between blood‐oxygen level dependent (BOLD) signal activations and respiration volume per time (RVT). Significance testing of coherence magnitude was p [file HBM-44-6537-s001.docx]

**
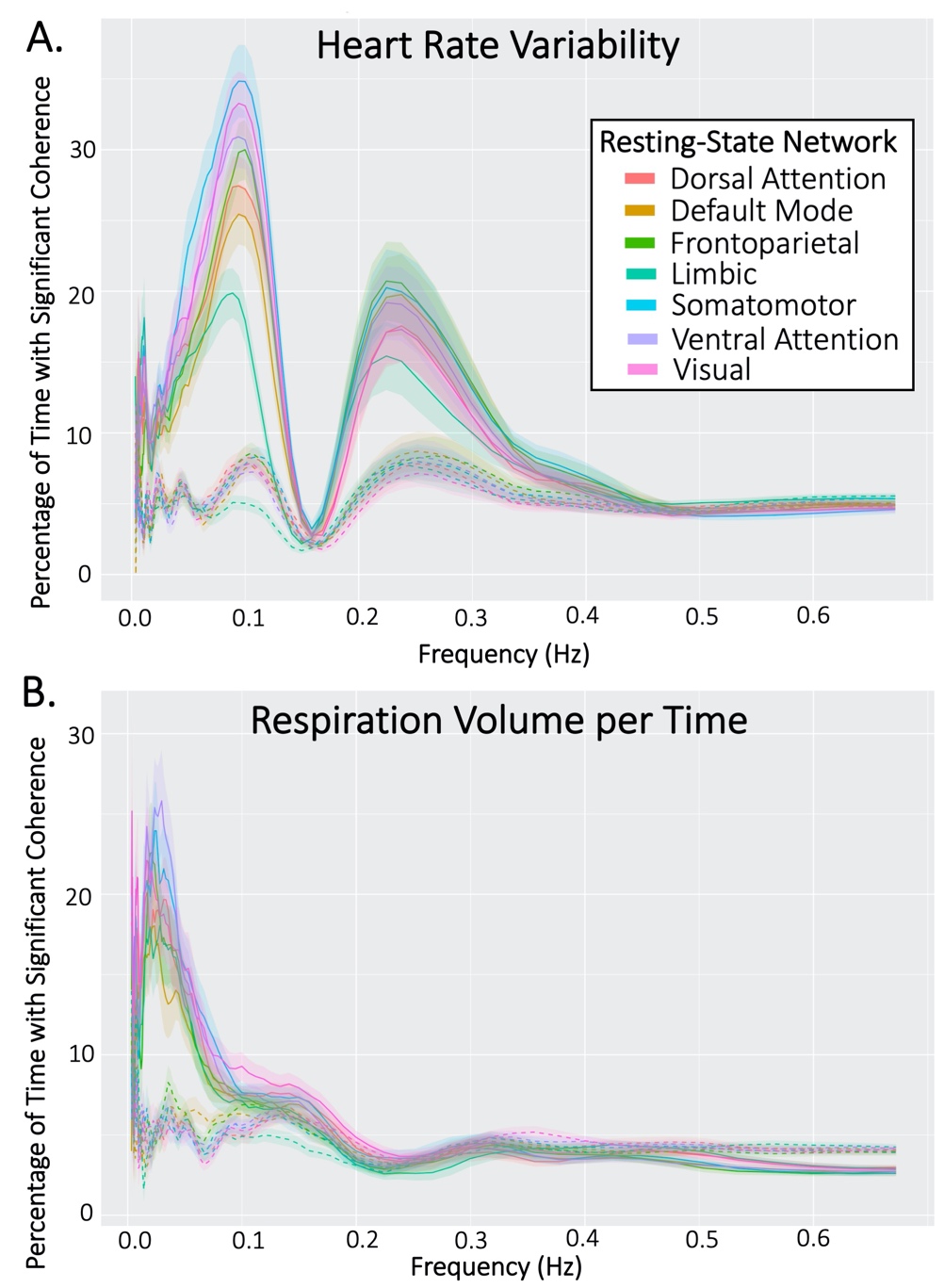
Supplementary Figures**

***Supplementary Figure 1.*** *Percentages of time with significant coherence between blood-oxygen level dependent (BOLD) signal activations and each systemic physiological dynamic (****A.*** *heart rate variability [HRV] and* ***B.*** *respiration volume per time [RVT]) were averaged across participants for each frequency and resting-state network (RSN). Coherence between RSN BOLD activations and a null HRV/RVT signal, with the same power spectrum as true HRV/RVT, are represented by dashed lines. Shaded areas represent standard error.*

**
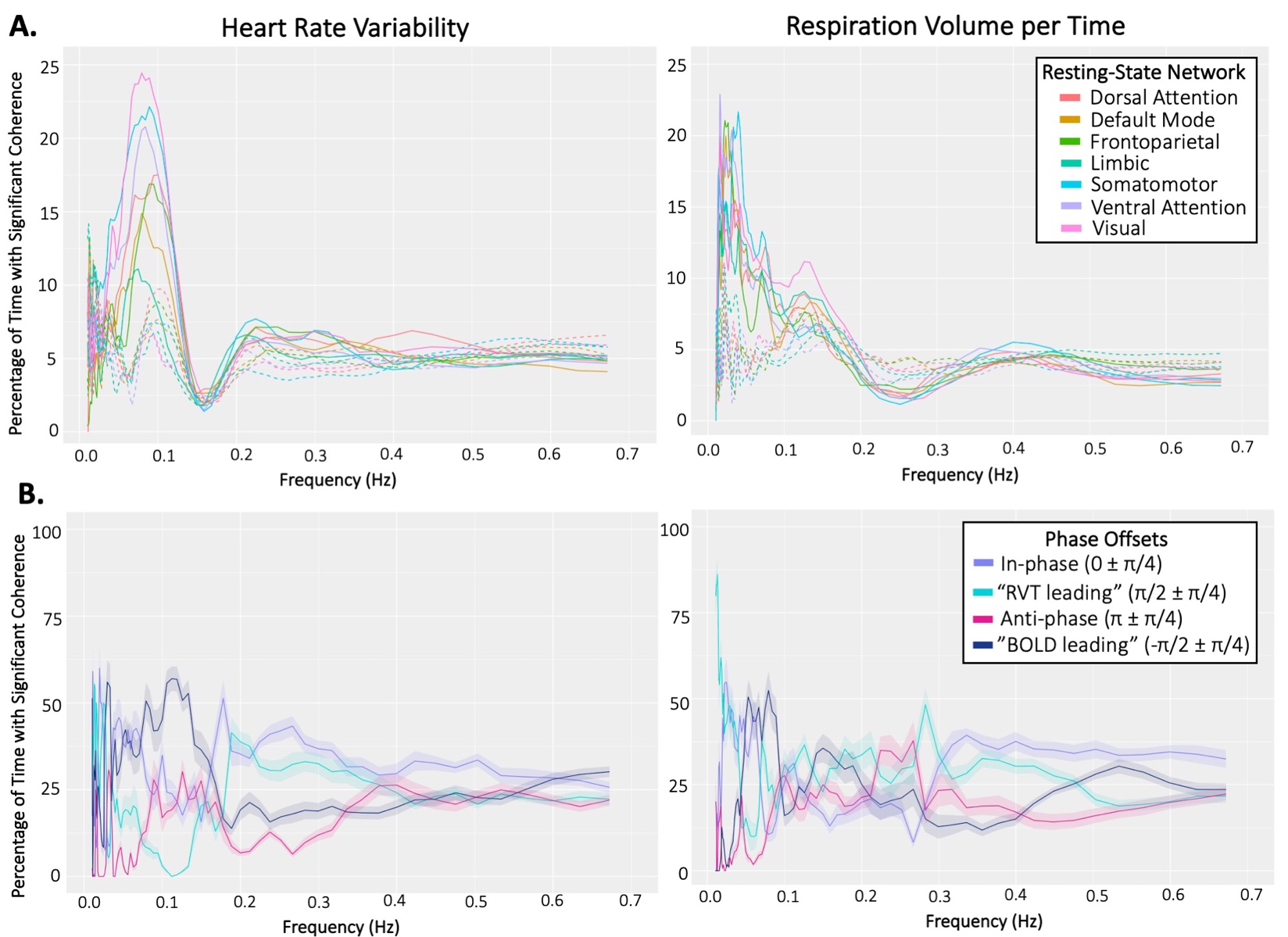
**

***Supplementary Figure 2****.* ***Coherence profiles after ICA-FIX denoising to remove quasiperiodic fluctuations in 10 participants. A.*** *Percentages of time with significant coherence between blood-oxygen level dependent (BOLD) signal activations in the default mode network (DMN) and each systemic physiological dynamic were averaged across participants for each frequency. Coherence between DMN BOLD activations and a null HRV/RVT signal, with the same power spectrum as true HRV/RVT, are represented by dashed lines. Shaded areas represent standard error.* ***B.*** *Phase offsets demonstrate distinct frequency profiles across networks. Shaded areas represent standard error.*


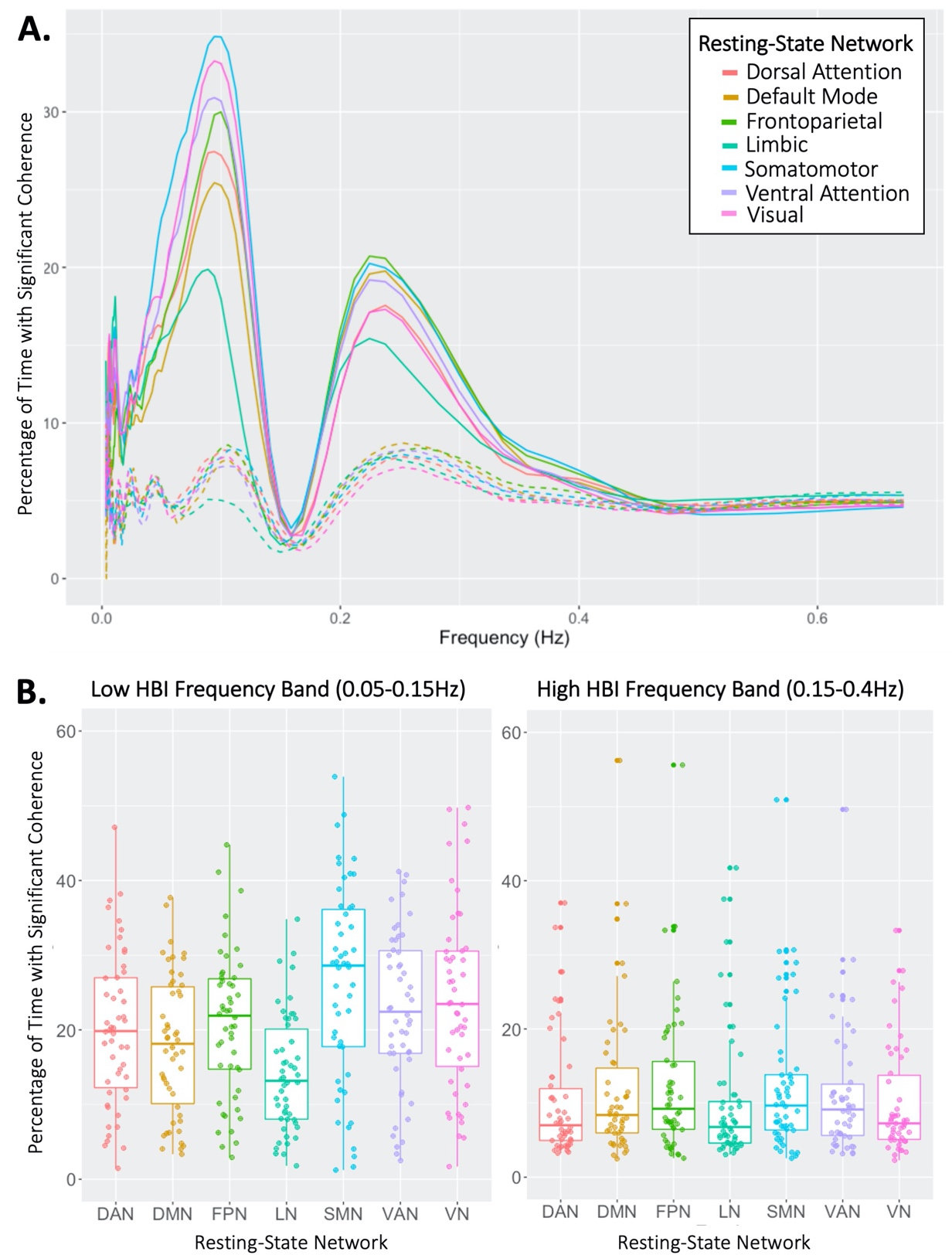


***Supplementary Figure 3.*** *Percentages of time with significant coherence between blood-oxygen level dependent (BOLD) signal activations and heartbeat interval (HBI). Significance testing of coherence magnitude was performed with a Monte Carlo approach with bootstrapped timeseries. BOLD signals and HBI were modeled using an autoregressive models of orders 1 and 9 respectively. After estimating AR1 coefficients for each signal, 300 pairs of bootstrapped timeseries were generated. WTC magnitude was then calculated for each bootstrapped pair to generate a null distribution and determine a 95% significance threshold at each scale (i.e. frequency).*


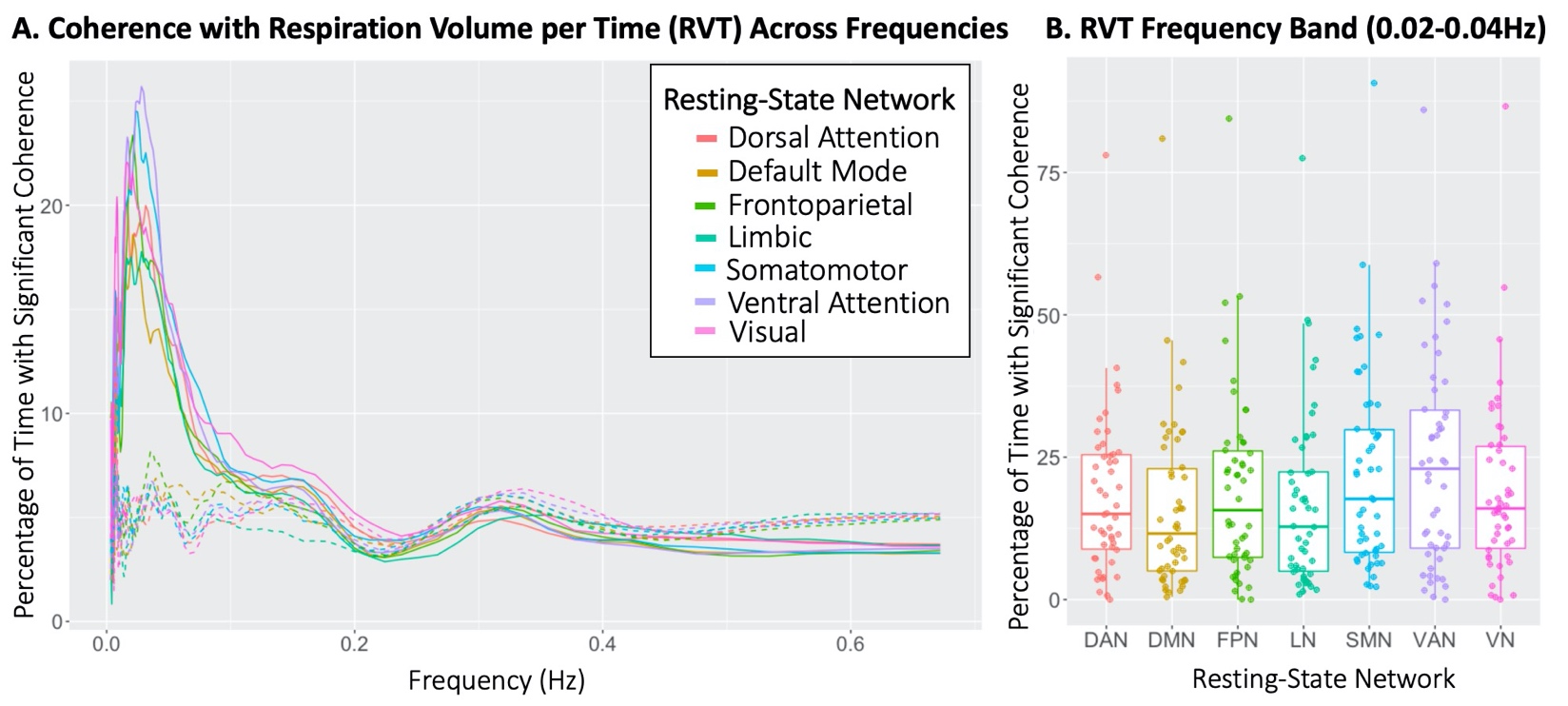


***Supplementary Figure 4.*** *Percentages of time with significant coherence between blood-oxygen level dependent (BOLD) signal activations and respiration volume per time (RVT). Significance testing of coherence magnitude was performed with a Monte Carlo approach with bootstrapped timeseries. BOLD signals and RVT were modeled using an autoregressive models of orders 1 and 9 respectively. After estimating AR1 coefficients for each signal, 300 pairs of bootstrapped timeseries were generated. WTC magnitude was then calculated for each bootstrapped pair to generate a null distribution and determine a 95% significance threshold at each scale (i.e. frequency).*

|  | | Low Frequency HRV  (0.05-0.15Hz) | | | High Frequency HRV  (0.15-0.4Hz) | | | RVT Frequency  (0.02-0.04Hz) | | |
| --- | --- | --- | --- | --- | --- | --- | --- | --- | --- | --- |
| RSN 1 | **RSN 2** | **t-value** | **p-value** | **Sig**† | **t-value** | **p-value** | **Sig**† | **t-value** | **p-value** | **Sig**† |
| DAN | **DMN** | 2.19 | 3.30E-02 | ns | -2.61 | 0.01 | ns | 1.74 | 8.80E-02 | ns |
| DAN | **FPN** | -0.94 | 3.50E-01 | ns | -3.22 | 2.00E-03 | * | -0.48 | 6.33E-01 | ns |
| DAN | **LN** | 5.49 | 1.43E-06 | **** | 0.55 | 0.58 | ns | 1.19 | 2.40E-01 | ns |
| DAN | **SMN** | -5.54 | 1.20E-06 | **** | -3.96 | 2.45E-04 | ** | -2.88 | 6.00E-03 | ns |
| DAN | **VAN** | -2.91 | 5.00E-03 | ns | -2.12 | 0.04 | ns | -4.15 | 1.31E-04 | ** |
| DAN | **VN** | -5.20 | 3.89E-06 | **** | 0.24 | 0.81 | ns | -0.50 | 6.22E-01 | ns |
| DMN | **FPN** | -3.51 | 9.70E-04 | * | -1.41 | 0.16 | ns | -3.25 | 2.00E-03 | * |
| DMN | **LN** | 3.90 | 2.94E-04 | ** | 3.02 | 4.00E-03 | ns | -0.97 | 3.35E-01 | ns |
| DMN | **SMN** | -6.03 | 2.12E-07 | **** | -0.76 | 0.45 | ns | -3.72 | 5.08E-04 | * |
| DMN | **VAN** | -4.37 | 6.49E-05 | ** | 1.17 | 0.25 | ns | -4.24 | 9.94E-05 | ** |
| DMN | **VN** | -4.41 | 5.68E-05 | ** | 2.08 | 0.04 | ns | -2.28 | 2.70E-02 | ns |
| FPN | **LN** | 6.52 | 3.70E-08 | **** | 3.55 | 8.72E-04 | * | 2.06 | 4.40E-02 | ns |
| FPN | **SMN** | -4.39 | 5.98E-05 | ** | 0.09 | 0.93 | ns | -2.01 | 5.00E-02 | ns |
| FPN | **VAN** | -2.07 | 4.40E-02 | ns | 2.21 | 0.03 | ns | -3.16 | 3.00E-03 | ns |
| FPN | **VN** | -3.16 | 3.00E-03 | ns | 2.61 | 0.01 | ns | -0.02 | 9.83E-01 | ns |
| LN | **SMN** | -7.92 | 2.50E-10 | **** | -2.87 | 0.01 | ns | -4.09 | 1.58E-04 | ** |
| LN | **VAN** | -7.24 | 2.78E-09 | **** | -2.01 | 0.05 | ns | -4.71 | 2.07E-05 | *** |
| LN | **VN** | -7.15 | 3.88E-09 | **** | -0.28 | 0.78 | ns | -1.54 | 1.31E-01 | ns |
| SMN | **VAN** | 3.66 | 6.11E-04 | * | 2.63 | 0.01 | ns | -1.54 | 1.30E-01 | ns |
| SMN | **VN** | 1.89 | 6.50E-02 | ns | 2.55 | 0.01 | ns | 2.26 | 2.90E-02 | ns |
| VAN | **VN** | -1.51 | 1.38E-01 | ns | 1.49 | 0.14 | ns | 3.03 | 4.00E-03 | ns |

***Supplementary Table 1.*** *Results of post-hoc paired t-tests comparing percent time with significant coherence between resting-state networks (RSN) in each frequency band. Significance testing of coherence magnitude was performed with a Monte Carlo approach with bootstrapped timeseries. BOLD signals and RVT were modeled using an autoregressive models of orders 1 and 9 respectively. After estimating AR1 coefficients for each signal, 300 pairs of bootstrapped timeseries were generated. WTC magnitude was then calculated for each bootstrapped pair to generate a null distribution and determine a 95% significance threshold at each scale (i.e. frequency).*

*Dorsal attention network (DAN), default mode network (DMN), frontoparietal network (FPN), limbic network (LN), somatomotor network (SMN), ventral attention network (VAN), visual network (VN). Significance adjusted with Bonferroni correction (Sig†).*


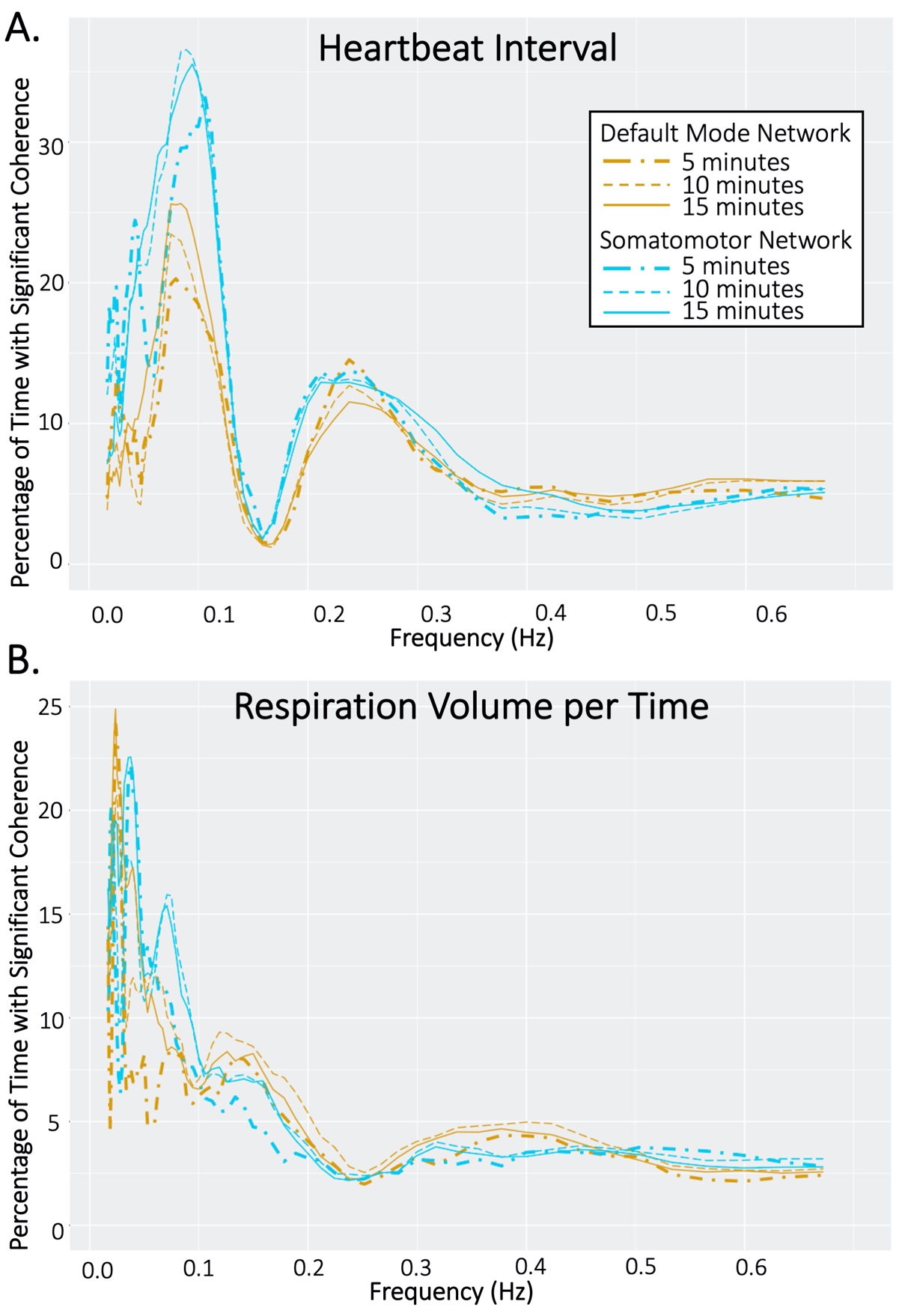


***Supplementary Figure 5. Coherence between systemic physiological dynamics and resting-state network (RSN) blood oxygen level dependent (BOLD) signal activations across scan durations.*** *The original 15-minute resting-state functional MRI scan was truncated into 5- and 10-minute segments to assess the reproducibility of our results across scan duration. Wavelet frequencies with greater than 2 minutes of scan time outside of the unreliable “cone of influence”, in which edge artifacts are problematic, are included.*
